# Research on Inter-Frame Feature Mismatch Removal Method of VSLAM in Dynamic Scenes

**DOI:** 10.3390/s24031007

**Published:** 2024-02-04

**Authors:** Zhiyong Yang, Yang He, Kun Zhao, Qing Lang, Hua Duan, Yuhong Xiong, Daode Zhang

**Affiliations:** 1 Engineering Research and Design Institute of Agricultural Equipment, Hubei University of Technology, Wuhan 430068, China; yzy017@126.com; 2 Hubei Engineering Research Center for Intellectualization of Agricultural Equipment, Wuhan 430068, China; 3 School of Mechanical Engineering, Hubei University of Technology, Wuhan 430068, China; maxhe1020@163.com (Y.H.);; 4 Hubei Key Laboratory Modern Manufacturing Quality Engineering, School of Mechanical Engineering, Hubei University of Technology, Wuhan 430068, China

**Keywords:** VSLAM, feature matching, GMS, improved RANSAC

## Abstract

Visual Simultaneous Localization and Mapping (VSLAM) estimates the robot’s pose in three-dimensional space by analyzing the depth variations of inter-frame feature points. Inter-frame feature point mismatches can lead to tracking failure, impacting the accuracy of the mobile robot’s self-localization and mapping. This paper proposes a method for removing mismatches of image features in dynamic scenes in visual SLAM. First, the Grid-based Motion Statistics (GMS) method was introduced for fast coarse screening of mismatched image features. Second, an Adaptive Error Threshold RANSAC (ATRANSAC) method, determined by the internal matching rate, was proposed to improve the accuracy of removing mismatched image features in dynamic and static scenes. Third, the GMS-ATRANSAC method was tested for removing mismatched image features, and experimental results showed that GMS-ATRANSAC can remove mismatches of image features on moving objects. It achieved an average error reduction of 29.4% and 32.9% compared to RANSAC and GMS-RANSAC, with a corresponding reduction in error variance of 63.9% and 58.0%, respectively. The processing time was reduced by 78.3% and 38%, respectively. Finally, the effectiveness of inter-frame feature mismatch removal in the initialization thread of ORB-SLAM2 and the tracking thread of ORB-SLAM3 was verified for the proposed algorithm.

## 1. Introduction

SLAM for mobile robots refers to the process of acquiring current environmental information through sensors such as cameras and LiDAR [1], tracking key features such as points, lines, and surfaces in the environment, and analyzing the depth changes of these key features, through which it achieves the simultaneous positioning of a mobile robot and mapping of the surrounding environment. The ability to effectively track key features in the environment is crucial for determining the accuracy of a mobile robot’s self-positioning and the quality of the surrounding environmental map building. Cameras capture rich environmental image information and are relatively cost-effective, making them the current mainstream sensors for SLAM [2]. Visual SLAM achieves feature point tracking through inter-frame feature point matching. The fast, accurate, and robust matching of image feature points in dynamic scenes poses a significant challenge for visual SLAM.

Andrew Davison et al. proposed the monocular camera visual SLAM algorithms MonoSLAM [3] and PTAM [4]. MonoSLAM achieves sensor pose estimation and surrounding environment 3D map construction by triangulating the matched feature points. Building on MonoSLAM, PTAM parallelizes the processing of pose estimation and map establishment, improving the real-time performance of MonoSLAM. SVO [5] combines direct methods, optical flow methods, and feature point methods to improve its own localization and surrounding environment mapping efficiency. Ethan et al. [6] propose the ORB feature by combining FAST (Features from Accelerated Segment Test) and BRIEF (Binary Robust Independent Elementary Features). ORB, with features like rotation invariance, ease of storage, and fast detection, is suitable for high real-time performance in visual SLAM systems. Based on this, ORB-SLAM [7] uses Oriented FAST and Rotated BRIEF (ORB) for image feature detection, improving the real-time performance and robustness of the front end of the SLAM system. Enhanced methods ORB-SLAM2 [8] and ORB-SLAM3 [9] are adaptable to multiple visual sensors. All the methods mentioned above involve feature point matching in visual odometry. Due to factors such as the presence of numerous similar textures and object occlusions in images, avoiding the occurrence of feature point mismatch is challenging [10], especially in dynamic scenes where a higher number of dynamic features can result in more mismatches. Simultaneously, the processing speed of feature point matching directly affects the real-time performance of visual SLAM methods. Improving the quality and speed of image feature mismatching is crucial for enhancing the performance of visual SLAM [11].

To address the aforementioned issues, this paper makes the following contributions:

- Propose an adaptive RANSAC method (ATRANSAC) capable of dynamically adjusting the image feature matching error threshold. Through a certain number of iterations, it identifies the minimum error threshold that satisfies the given inlier matching rate. This method dynamically adjusts the error threshold with relatively fewer iterations compared to traditional RANSAC methods, providing a precise and fast image feature mismatch removal approach.
- The GMS method [12] has a fast detection speed and offers relatively reliable filtering of image feature-matching results. The combination of GMS and ATRANSAC methods effectively removes image feature mismatches. The GMS coarse screening set is downsampled to reduce the computational burden of ATRANSAC, facilitating the identification of the optimal set of image feature matches with very few iterations.
- Conduct detailed testing on the performance and feasibility of GMS-ATRANSAC. First, this proposed method was visually compared to the classical method RANSAC and the latest methods GMS and GMS-RANSAC for image feature matching effects in various scenes within the TUM [13] and KITTI [14] datasets. Subsequently, a comparative analysis of the accuracy and processing speed in the mismatch removal process for the mentioned methods was conducted in dynamic environments. Finally, the proposed method was applied to the initialization thread of ORB-SLAM2 and the ORB-SLAM3 tracking thread for feasibility verification.

In Section 2, this paper elaborates on the relevant work of image mismatch removal algorithms. Section 3 provides a detailed description of the algorithm proposed in this paper, with a comprehensive explanation of algorithm details. Section 4 constitutes the experimental part of this paper, designing evaluation metrics and conducting detailed experiments on public datasets. This section also includes a comparison with GMS, RANSAC, and GMS-RANSAC. Subsequently, the proposed algorithm was validated in both ORB-SLAM2 and ORB-SLAM3. Section 5 summarizes the work undertaken in this paper and provides an outlook on future improvement efforts.

## 2. Related Works

Random Sample Consensus (RANSAC) is a widely used robust fitting method [15]. The core idea is to calculate a fitting model by randomly sampling the observed data, evaluate the fitting quality through the residuals between the fitting model and all observed data points, and iteratively search for the optimal fit. Fischler et al. [16] used RANSAC to remove mismatched feature points in image feature matching. They randomly selected four pairs of matching points in images $P_{A}$ and $P_{B}$. Then, they used the Direct Linear Transform (DLT) method to solve for the homography matrix *H*.

(1) $$c\;\left(\begin{array}{c} u\\ v\\ 1 \end{array}\right)=H\;\left(\begin{array}{c} u^{\prime }\\ v^{\prime }\\ 1 \end{array}\right)$$

In Equation (1), where *c* is a constant, ${p=\left(\begin{array}{ccc} u & v & 1 \end{array}\right)}^{t}$ and p is a point in image $P_{A}$, ${p^{\prime }=\left(\begin{array}{ccc} u^{\prime } & v^{\prime } & 1 \end{array}\right)}^{t}$, $p^{\prime }$ is a point in image $P_{B}$, p and $p^{\prime }$ form a matching pair, and $H=\left(\begin{array}{ccc} h_{1} & h_{2} & h_{3}\\ h_{4} & h_{5} & h_{6}\\ h_{7} & h_{8} & h_{9} \end{array}\right)$.

After obtaining the homography matrix *H*, the matched feature points $K_{P}^{A}=\{k_{p1}^{A},k_{p2}^{A},...k_{pn}^{A}\}$ in image $P_{A}$ are reprojected into $P_{B}$ according to Equation (1), resulting in the reprojection point set $K_{P}^{AB}=\{k_{p1}^{AB},k_{p2}^{AB},...k_{pn}^{AB}\}$. Subsequently, based on Equation (2), the reprojection error *e* is calculated by taking the differences between the reprojection point set $K_{P}^{AB}$ and the feature point set $K_{P}^{B}=\{k_{p1}^{B},k_{p2}^{B},...k_{pn}^{B}\}$ in image $P_{B}$.

(2) $$e=||k_{pi}^{AB}-k_{pi}^{B}||$$

If the obtained reprojection error *e* is less than the given error threshold, the current point is considered an inlier, and the suitability of the current homography matrix is judged based on the accumulated inlier count. Subsequently, random point sampling is continuously performed to iteratively update the optimal homography matrix *H*. It is worth mentioning that Hartley et al. [17], based on prior knowledge, specified an inlier rate and calculated the probability that at least one iteration could achieve this inlier rate. This probability was then used to deduce the optimal number of iterations. In current RANSAC usage, both the error threshold and the number of iterations are fixed values. Excessive iteration numbers impact algorithm efficiency, and a fixed error threshold results in less accurate fitting models. Since the proposal of RANSAC, many scholars have conducted research on the improvement of RANSAC, including improvements in accuracy, efficiency, and robustness. For accuracy improvement, Torr et al. [18] proposed a variant of RANSAC called MLESAC based on maximum likelihood estimation. It achieves more accurate fitting by iteratively finding optimal model parameters and computing the likelihood probability of data points through random sample points. The complex calculation of likelihood probability for random samples leads to lower efficiency of the algorithm. Chum et al. [19] proposed LO-RANSAC, which introduces a local optimization mechanism after each iteration to improve the fitting accuracy and robustness of local models. Zhang et al. [20] improved traditional camera calibration methods based on LO-RANSAC to achieve automatic acquisition of the best calibration results. However, LO-RANSAC exhibits poor global consistency in the presence of large scene changes. Barath et al. [21] proposed GC-RANSAC based on LO-RANSAC, which segments images during local optimization to enhance global consistency. However, the complexity of the image segmentation algorithm limits real-time performance. Efficiency improvement includes methods based on guided sampling and partial evaluation, such as the guided sampling-based method PROSAC [22]. This method prioritizes samples based on prior information, randomly samples from the subset of data points with the highest evaluation function values, and performs model fitting. Xin et al. [23] applied PROSAC to indoor mobile robot visual SLAM. Due to PROSAC’s guided sampling feature, its robustness is poor in dynamic scenes. NAPSAC [24] and GASAC [25] do not utilize prior information to handle the sample set. NAPSAC uses a heuristic method, assuming that inlier points are closer to other inlier points, to sample data within a defined radius starting from randomly sampled points. It performs well in fitting data models with high noise. GASAC is a sampling consensus method based on genetic algorithms. It generates and optimizes hypothesis models by evolving subsets of the dataset as genes, evaluates fitness, and improves fitting effectiveness. However, guided sampling limits global search and cannot adapt to scenes with significant changes. Partial evaluation is conducted during random sampling in R-RANSAC [26]. If the evaluation result is poor, the next sampling is performed to reduce computation time. However, this method relies on testing and cannot adapt to changing scenes. To improve robustness, MAPSAC [27] introduces probability models and a Bayesian framework. It fits models and detects outliers through maximum a posteriori probability estimation. Xu et al. [28] applied MAPSAC in outdoor mobile robot navigation with distant scenes and repeated structures, but the high computational complexity resulted in low algorithm efficiency and poor real-time performance. This paper proposes an adaptive error threshold RANSAC method. This method uniformly downsamples the sample set before iteration and rapidly determines the internal matching set corresponding to the minimum error threshold based on the internal matching rate. While considering environmental adaptability, it enhances algorithm efficiency and more accurately estimates the fitting model.

GMS (Grid-based Motion Statistics) is a grid-based motion analysis technique that assumes under smooth motion; correct feature points will have more surrounding matching feature points. The GMS method divides the image into several equally sized grid cells, performs statistical analysis on grid units with moving pixels, reduces the algorithm’s time complexity, and quickly obtains more feature matches in dynamic scenes. Lan et al. [29] and Zhang et al. [30] integrated GMS with RANSAC and applied it to UAV image stitching and visual SLAM, respectively. Through GMS coarse screening, they appropriately reduced the number of RANSAC iterations, improving the algorithm’s speed. However, due to the fixed error threshold of the algorithm, it cannot achieve more accurate matching in dynamic scenes.

## 3. Methodology

### 3.1. GMS-ATRANSAC Framework

Three-dimensional visual SLAM (3D VSLAM) based on the feature point method captures two-dimensional images of the surrounding environment through a camera, treating each image as a frame. Visual odometry uses image feature matching to track the position changes of the same feature points in different frames, estimate the sensor pose, and construct a map of the surrounding environment. The accuracy and speed of removing image feature mismatches directly impact the effectiveness of SLAM. This paper proposes a method to combine GMS with the adaptive error threshold RANSAC (GMS-ATRANSAC) for removing inter-frame feature mismatches in 3D VSLAM. This method enables more accurate and faster tracking of inter-frame feature points in dynamic scenes of 3D VSLAM.

**Figure 1:**
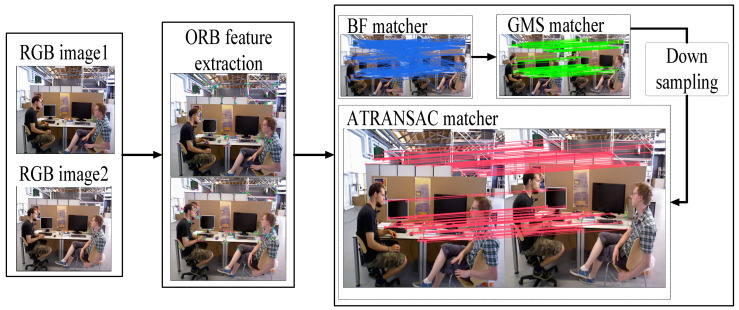
The workflow of GMS-ATRANSAC; the images are derived from the TUM [22] dataset.

ORB features are used for 2D image processing. It combines FAST keypoints with BRIEF descriptors. The FAST keypoint extraction method efficiently and accurately identifies areas with significant texture variations. The BRIEF descriptor method compares each FAST keypoint with surrounding pixels and uses a binary string to describe the local image structure of the keypoint. These features give ORB advantages such as rotation invariance, ease of storage, and fast detection, making it more efficient than feature extraction methods like SIFT and SURF [6]. This benefits the accuracy and real-time performance of feature extraction and tracking in 3D VSLAM. In the following sections, ORB is used as image feature points in the GMS-ATRANSAC process. The workflow of GMS-ATRANSAC is shown in Figure 1, and the process is as follows:

1. Capture continuous 2D images *P_A_* and *P_B_* through the camera.
2. Extract FAST keypoints in images *P_A_* and *P_B_*.
3. Calculate the BRIEF descriptors corresponding to the FAST keypoints in images *P_A_* and *P_B_*, completing the ORB feature extraction.
4. Obtain a matching set $S_{BF}$ by using brute-force matching [31] based on Hamming distance for ORB features.
5. Apply the GMS method to perform coarse screening on the brute-force matching set $S_{BF}$, obtaining the coarse matching set $S_{GMS}$.
6. Downsample the coarse screening matching set $S_{GMS}$ to obtain the downsampled matching set $S_{DS}$.
7. Apply the ATRANSAC algorithm proposed in this paper for fine screening in the downsampled matching set $S_{DS}$

### 3.2. Adaptive Threshold RANSAC

Traditional RANSAC is a robust fitting method widely used in removing mismatches in image feature matching. The traditional RANSAC method requires pre-setting of error threshold and iteration times. Due to the randomness of RANSAC, with an infinite number of iterations, traditional RANSAC can obtain an optimal matching result. However, in a limited number of iterations, after reaching the set maximum iteration times, the obtained matching result may not be optimal. To make RANSAC more adaptive, generally, a larger error threshold is set. The randomness of a limited number of iterations and a larger error threshold cause RANSAC not to be consistently accurate, and a higher number of iterations requires more time. This paper proposes an Adaptive Threshold RANSAC (ATRANSAC) method determined by the internal matching rate. This method introduces the minimum internal matching rate *P_min_* and iteratively searches for the minimum error threshold corresponding to *P_min_*.

Similar to RANSAC, ATRANSAC calculates the reprojection error $e_{i}$ for each point based on the computed homography matrix *H*. However, in ATRANSAC, the reprojection error $e_{i}$ for each point is compared with the current iteration’s error threshold $e_{t}$.

(3) $$\left\{\;\begin{array}{c} \;\;m_{i}\in M_{n},e_{i}\leq e_{t}\;\\ {m_{i}\cap M_{n}=\emptyset ,e}_{i}>e_{t} \end{array}\right.$$

In Equation (3), $m_{i}$ represents the match between the feature point $k_{pi}^{A}$ in image *P_A_* and the feature point $k_{pi}^{B}$ in image *P_B_*. If $e_{i}\leq e_{t}$, $m_{i}$ is added to the internal matching set *M_n_*. After completing the calculation of reprojection errors for all matched feature points, the internal matching rate is calculated using the following equation:

(4) $$P=\frac{Q}{N}$$

In Equation (4), *Q* is the number of elements in the internal matching set, *N* is the total number of matches, and *P* is the internal matching rate for the current iteration. If $P\leq P_{min}$, the internal matching set for the current iteration represents the most accurate matches. The specific algorithmic process is as follows:

1. Set the initial maximum internal matching rate *P_max_*, minimum internal matching rate *P_min_*, error threshold *e_t_*, and matching set $S_{m}=\{K_{P}^{A},K_{P}^{B},M_{AB}\}$
2. Randomly extract four matches from the matching sample set and calculate the homography matrix *H*.
3. Calculate the reprojection point set $K_{P}^{AB}=\{k_{p1}^{AB},k_{p2}^{AB},...k_{pn}^{AB}\}$ by projecting the matching point set from image *P_A_* to image *P_B_* based on the homography matrix *H*.
4. Calculate the error for each matching point using Formula (2) and determine whether it is an internal matching using Equation (3); if it is an internal matching, add it to the internal matching set *M_n_* and record the internal matching count *Q*.
5. Check if the current iteration’s internal matching count *Q* is less than 4. If it is less than or equal to 4, output the previous internal matching set *M_n−_*_1_. If *Q* is greater than 4, proceed to the next step.
6. Calculate the internal matching rate *P* using Equation (4). If $P>P_{max}$ update the error threshold *e_t_* to *α* times its original value (where *α* < 1) and repeat from step (2). If $P\leq P_{max}$, reduce $P_{max}$ by *β*.
7. If $P_{max}\leq P_{min}$, output the current internal matching set *M_n._*

**Figure 2:**
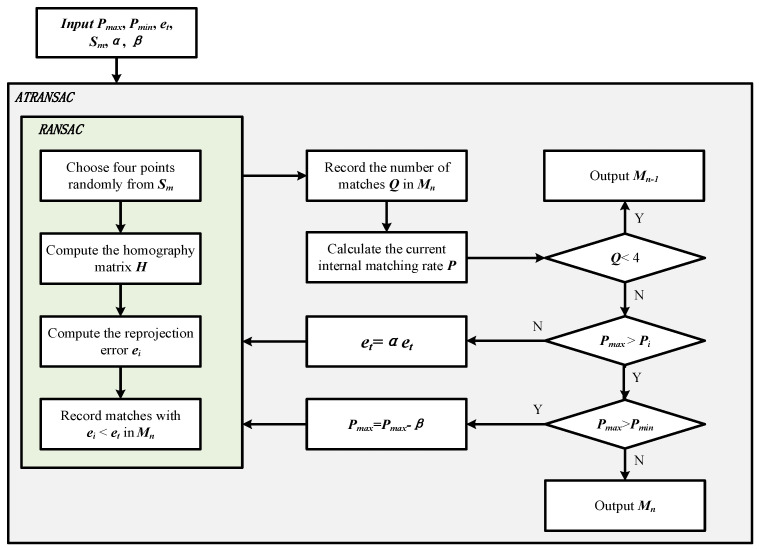
The workflow of ATRANSAC.

ATRANSAC iteratively reduces the error threshold based on the internal matching rate, finding the smallest error threshold under the current given internal matching rate, i.e., the most accurate homography. This algorithm relies on prior parameter settings, and parameters such as initial error threshold $e_{t}$, maximum internal matching rate *P_max_*, and adjustment factor *β* play a crucial role in the convergence of iterations. To ensure that ATRANSAC effectively converges in different scenarios, an explanation of parameter settings is provided. Since the method’s error threshold is adaptively reduced, the initial error threshold $e_{t}$ should be set relatively large. If the initial error threshold $e_{t}$ is too small, the number of internal matches obtained may be too few, failing to meet the initial minimum internal matching rate, which affects the robustness of the algorithm. To prevent situations in specific environments, such as low lighting conditions or extremely low-texture scenarios, where there may not be enough sets of image feature matches to calculate the homography matrix *H*, in step 5 of the algorithm flow, it is necessary to check the number of internal matches. If the number of internal matches is too small, the parameters for calculating the transformation matrix are insufficient, leading to poor robustness of the algorithm. Hence, it is essential to maintain the number of internal matches above four. *α* is the error threshold coefficient, and its value determines the accuracy and efficiency of the algorithm. If the chosen value for *α* is too large, although the threshold updates more accurately, the algorithm’s iteration count is high, affecting the execution speed. If *α* is chosen to be small, the algorithm’s iteration count is low, resulting in a greater speed but lower accuracy. Generally, *α* is chosen between 0.8 and 0.95. $P_{max}$ also affects the algorithm’s iteration count. In general, $P_{max}$ can be set between 0.7 and 0.9. Setting a higher value for the maximum internal matching rate $P_{max}$ will increase the algorithm’s iteration count and decrease efficiency. Since the RANSAC process is stochastic, if $P_{max}$ is too small, it may lead to instability in the algorithm and result in poorer image feature matching results. *β* can be set between 0.05 and 0.1, and a smaller *β* value can make the algorithm more stable. The algorithm balances accuracy and efficiency while ensuring convergence through three aspects: algorithm mechanism, parameter settings, and maintenance of internal matching numbers. The ATRANSAC flowchart is shown in Figure 2, and the pseudo code is shown in Algorithm 1.

Algorithm 1. Pseudo code of ATRANSAC.
**Algorithm 1:** Adaptive error threshold RANSAC**Input:** Sample set:     $S_{m}=\{K_{P}^{A},K_{P}^{B},M_{AB}\}$   Initial parameters:      Maximum internal matching rate *P_max_*     Minimum internal matching rate *P_min_*
     Error Threshold *e_t_*     Error Threshold Coefficient α     Maximum inlier ratio adjustment factor β **Output:** Internal matching set *M_n_*     1.Procedure ATRANSAC ($S_{m}$, *P_max_*, *P_min_*, *e_t_*):     2.**while** (P_max_ > P_min_):     3.Set *M_n_* = {}     4.Randomly extract four matches from $S_{m}$     5.Calculate *H* by using Equation (1)     6.Calculate the error for each matching by using Equation (2)     7.**if** (The match is internal match by using Equation (3))     8.   Put internal match in *M_n_*     9.   *Q* = count (*M_n_*)     10.   **if** (*Q* < 4):     11.     return *M_n-1_*     12.   **else**:     13.     Calculate internal matching rate *P* using Equation (4)     14.     **if** (*P* > *P_max_*):     15.        *e_t_* = α*e_t_*     16.        Continue     17.     **else**:     18.        *P_max_* = *P_max_* − *β*     19.        Continue     20.**return** *M_n_*

### 3.3. GMS-ATRANSAC for Image Feature Mismatch Removal

**Figure 3:**
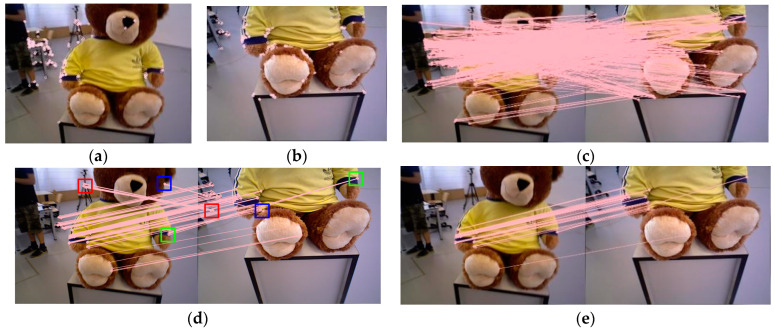
Using GMS-ATRANSAC for ORB feature matching on the TUM dataset, with 800 ORB features and a GMS grid size set to 20. (**a**), (**b**) show the extraction of ORB features from the images to be matched, (**c**) illustrates brute-force matching, (**d**) depicts GMS matching, and (**e**) shows ATRANSAC matching.

In the processing of image feature mismatches, both GMS and RANSAC possess unique advantages. Due to the motion smooth constraint introduced by GMS in removing mismatched feature matches and its grid processing, it obtains a larger number of matches and is very fast in dynamic scenes. However, it is sensitive to grid size setting and image occlusion, leading to the occurrence of mismatches. Due to the motion smooth constraint introduced by GMS in removing mismatched feature matches and its grid processing, it obtains a larger number of matches and is very fast in dynamic scenes. However, it is sensitive to grid size setting and image occlusion, leading to the occurrence of mismatches. RANSAC, in the process of removing mismatches, exhibits strong robustness, but the iteration count and fixed threshold limit the algorithm’s efficiency and accuracy. First, the ORB features of the images to be matched are extracted, as shown in Figure 3a,b. Second, brute-force matching is performed on the extracted features, as shown in Figure 3c. Subsequently, the GMS method is used for coarse screening of the pairs obtained from brute-force matching. Finally, ATRANSAC is used to conduct fine screening on the coarse screening set, as shown in Figure 3e, to obtain accurate matches.

After brute-force matching of image features, a large number of image feature pairs are generated, including both correct and mismatched pairs. Directly using RANSAC for filtering would lead to a large number of iterations and computational overhead for reprojection. Improper threshold settings may lead to either an excessive number of inaccurate matches or too few matches. Therefore, in this algorithm, using the GMS for coarse screening can rapidly filter out the majority of mismatched pairs, ensuring relatively accurate matches. Nonetheless, GMS is sensitive to grid size and image occlusion, and it still retains some mismatched or imprecise matches, as illustrated in Figure 3d. The mismatches identified by red and blue colors originate from image occlusion, while the imprecise matches marked in green are due to the gridded nature of the GMS method, where adjacent grids contain numerous similar features, resulting in some inaccuracies. Since the matching set obtained after coarse matching with the GMS is relatively accurate, using traditional RANSAC for optimization would lead to too many iterations, and a fixed threshold would result in matches that are still not precise enough. To achieve more accurate and faster image feature matching, the proposed ATRANSAC method is used for fine screening, employing adaptive error thresholding in a limited number of iterations, ultimately obtaining a precise set of image feature matches.

## 4. Experimental

The experimental environment consists of Ubuntu 18.04, a 4-thread, 6 GB RAM VMware virtual machine. The computer’s CPU is AMD Ryzen 5 5600G, and the algorithm is implemented in C++.

### 4.1. Dataset and Metrics

The proposed method GMS-ATRANSAC in this paper was visually compared to RANSAC [13], GMS [17], and GMS-RANSAC [19] for image feature mismatch removal effects in static, indoor dynamic, and outdoor dynamic scenes of the TUM and KITTI datasets. Additionally, a comparative analysis of mean error ($e_{mean}$), error variance ($e_{Var}$), and processing time during the mismatch removal process of the abovementioned methods was conducted to validate the accuracy and efficiency of the GMS-ATRANSAC method. The mean error represents the average size of all point error amounts, as shown in Formula (5).

(5) $$e_{mean}=\frac{\sum_{i=1}^{n}\left||k_{pi}^{AB}-k_{pi}^{B}|\right|}{n}$$

(6) $$e_{Var}=\frac{\sum_{i=1}^{n}{{(e}_{mean}-||k_{pi}^{AB}-k_{pi}^{B}||)}^{2}}{n}$$

Equation (6) expresses the error variance, indicating the dispersion of each matching error, providing insights into the algorithm’s fitting performance.

(7) $$Imprv(E)=\left(1-\frac{E_{our}}{E_{other}}\right)\times 100\%$$

In Equation (7), *E* represents the value of the metric. It can calculate the percentage improvement of various metrics between the proposed algorithm and other algorithms.

### 4.2. Removal of Image Feature Mismatches in Different Scenarios

**Figure 4:**
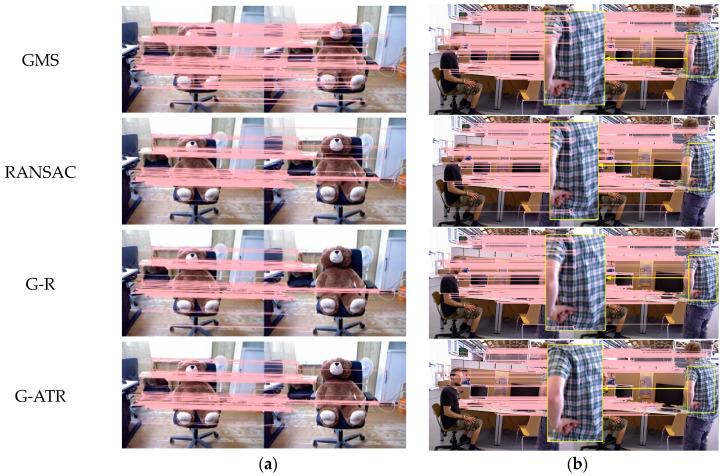
Comparison of the effects of mismatch removal for GMS [17], RANSAC [13], G-R (GMS-RANSAC [19]), and proposed method G-ATR (GMS-ATRANSAC) in static (**a**) and indoor dynamic scenes (**b**).

**Figure 5:**
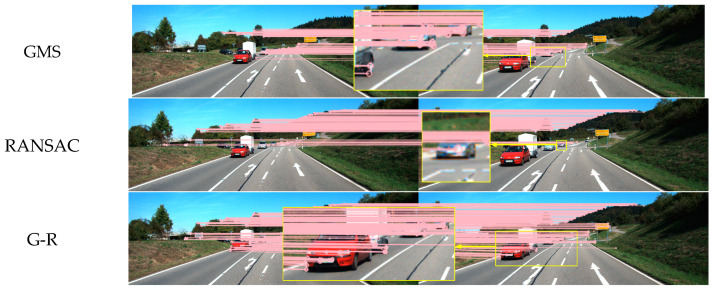

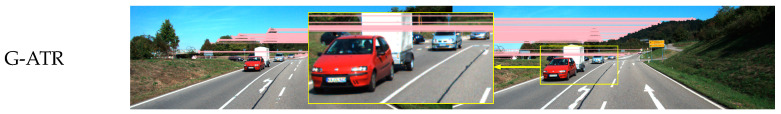
Comparison of mismatch removal effects for GMS, RANSAC, G-R (GMS-RANSAC), and G-ATR (GMS-ATRANSAC) in outdoor dynamic scenes.

As shown in Figure 4 and Figure 5, three experimental scenarios were set up: static, indoor dynamic, and outdoor dynamic. In Figure 4b, the indoor dynamic scene demonstrates the movement of people, such as walking and changes in gestures. The right side of the image is enlarged to showcase the performance of various algorithms in indoor dynamic scenes. Figure 5 presents the image feature matching effects of various algorithms in outdoor dynamic scenes with moving vehicles. In each scenario, 1000 ORB feature points were extracted. The GMS grid size parameter was set to the default value of 20. RANSAC had a threshold of three, with 50 iterations. In GMS-RANSAC, the RANSAC threshold remained unchanged, and the iteration count was set to 20 to improve algorithm speed. In GMS-ATRANSAC, the GMS parameters remained unchanged, and the internal matching rate was set to 40%. As shown in Figure 4a, all four algorithms perform well in static scenes, with GMS obtaining a large number of feature matches. In indoor dynamic scenes, as depicted in Figure 4b, GMS, RANSAC, and GMS-RANSAC retain many feature mismatches on the dynamically moving person, while GMS-ATRANSAC can remove feature mismatches related to the dynamic person in the image. As shown in Figure 5, due to the characteristic of GMS relying on the assumption of motion smoothness, GMS obtains many dynamic feature mismatches in outdoor dynamic scenes. In GMS-RANSAC, the randomness of RANSAC may result in fewer dynamic feature mismatches. The adaptive error threshold characteristic of GMS-ATRANSAC allows it to adaptively choose the minimum error threshold under a certain internal matching rate, accurately removing dynamic feature mismatches in dynamic scenes. These results indicate that GMS-ATRANSAC robustly filters dynamic feature mismatches in dynamic scenes.

**Figure 6:**
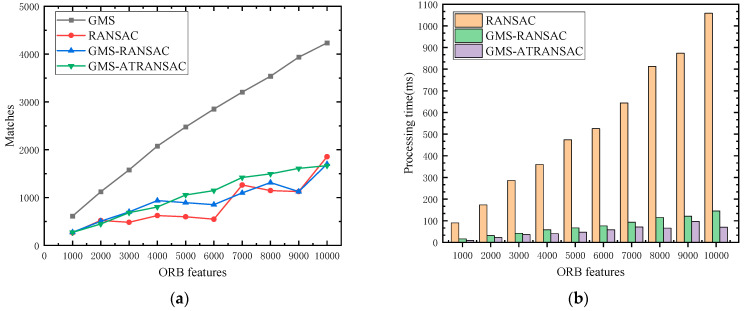
Comparison of match count (**a**) and processing time (**b**) for four algorithms under different feature point quantities.

Figure 6a illustrates the number of matches obtained by GMS, RANSAC, GMS-RANSAC, and GMS-ATRANSAC methods in processing different numbers of ORB feature points in outdoor dynamic scenes. All four methods can effectively remove mismatches. However, to enhance algorithm efficiency, selecting a smaller number of iterations for RANSAC and GMS-RANSAC results in a less linear increase in the number of high-quality matches as the number of feature points grows. If feature points continue to increase, the algorithm may reach a local optimum. GMS is relatively robust but struggles to remove dynamic matches in dynamic scenes. GMS-ATRANSAC achieves linear results and can remove a significant number of mismatches. Figure 6b shows the time spent by RANSAC, GMS-RANSAC, and GMS-ATRANSAC in removing mismatches in outdoor dynamic scenes. RANSAC, due to its higher iteration count and the need to traverse all feature points in each iteration, experiences a noticeable increase in time as the number of feature points grows. GMS-RANSAC and GMS-ATRANSAC methods consume less time, with GMS-ATRANSAC having the least time consumption due to uniform downsampling before ATRANSAC execution, significantly reducing processing time.

**Figure 7:**
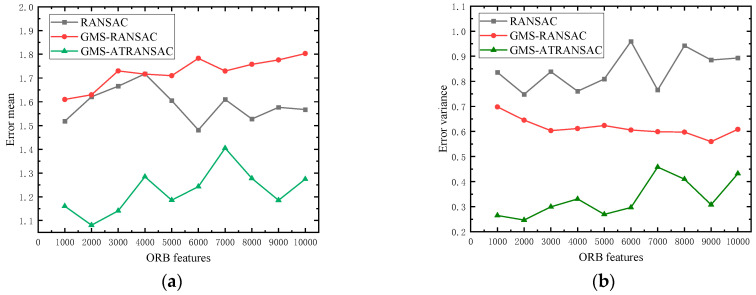
Comparison of the matching mean error (**a**) and error variance (**b**) of RANSAC, GMS-RANSAC and GMS-ATRANSAC at different feature points.

As shown in Figure 7a,b, GMS-ATRANSAC achieves smaller fitting mean error and error variance in matches with different numbers of feature points. This indicates that GMS-ATRANSAC provides a more accurate fit in image feature matching, and each set of matches has a better fit. It is a precise and robust algorithm for removing mismatches in image feature matching.

**Figure 8:**
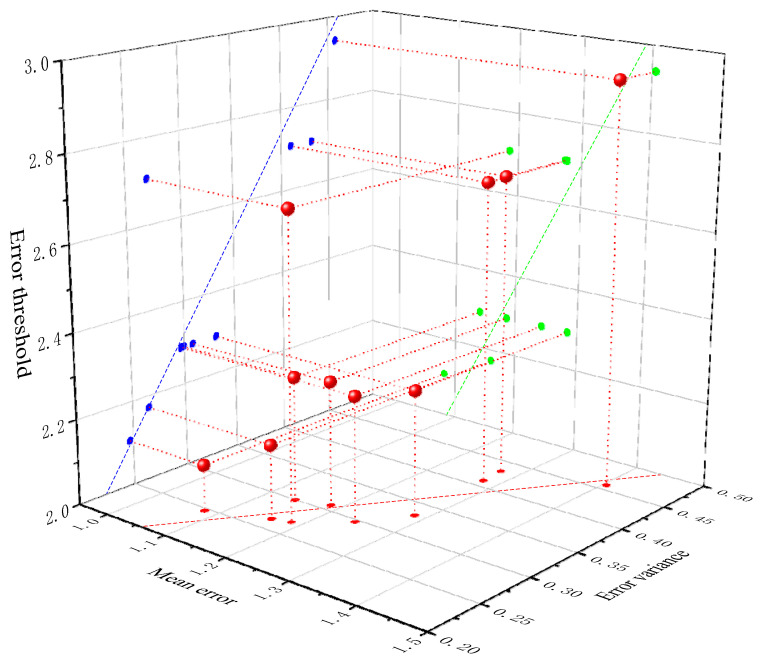
Polynomial fitting of the error threshold, $e_{mean}$, and $e_{Var}$ discrete points for the proposed algorithm in dynamic scenes.

Figure 8 clearly illustrates the relationship between error threshold, mean error, and error variance. First, the error threshold in outdoor dynamic scenes is represented on the Z-axis, the mean error on the X-axis, and the error variance on the Y-axis. This creates points in a 3D coordinate system. Subsequently, the spatial points are projected onto XY (red points), XZ (green points), and YZ (blue points). Finally, fits are performed within the three planes. The fit between the error threshold and error mean is represented by the green dashed line in the XZ plane, the fit between the error threshold and error variance is represented by the blue dashed line in the YZ plane, and the fit between mean error and error variance is represented by the red dashed line in the XY plane. From the fitted lines, it can be observed that there is a proportional relationship between error threshold, mean error, and error variance, meaning that a larger error threshold will lead to increased values of mean error and error variance. This also confirms the effectiveness of GMS-ATRANSAC in image feature mismatching removal. When the error threshold reaches its optimal value under the current internal matching rate, the error mean and error variance are minimized, resulting in high reprojection fitting, ultimately achieving accurate image feature matching.

Table 1 lists the mean error, error variance, and processing time of three algorithms in different datasets and environments. It can be seen that GMS-ATRANSAC outperforms the comparison algorithms in all three environments.

Table 2 presents a comparison of the mean error, error variance, and processing time between the proposed algorithm and other algorithms. In the comparison of fitting effects, GMS-ATRANSAC reduces the mean error by 29.4% and the error variance by 63.9% compared to RANSAC in indoor static, indoor dynamic, and outdoor dynamic scenes. Compared to GMS-RANSAC in three different scenarios, GMS-ATRANSAC reduces the mean error by 32.9% and the error variance by 58.0%. Moreover, compared to RANSAC and GMS-RANSAC, the processing time is reduced by 78.3% and 38.0%, respectively, making it a fast and accurate algorithm for image feature mismatch removal.

### 4.3. Inter-Frame Feature Mismatch Removal Based on GMS-ATRANSAC in ORB-SLAM

GMS-ATRANSAC was applied to image feature matching by filtering in the ORB-SLAM series algorithms. The experiments were conducted in monocular mode, and the effectiveness of GMS-ATRANSAC was verified in the Initializer thread of ORB-SLAM2 and the Tracking thread of ORB-SLAM3. The test environment used the rgbd_dataset_freiburg3_walking_xyz sequence from the TUM dataset.

#### 4.3.1. Initializer

The monocular initialization of ORB-SLAM2 is relatively complex, requiring a period of operation to find the optimal initial pose, which effectively demonstrates the importance of removing image feature mismatches. First, 1000 ORB feature points are extracted for each frame, and frame-to-frame feature matching is performed on the extracted ORB feature points. Second, the number of matching points is checked, and if it is less than 100, reinitialization is performed. If initialization is successful, depth values are estimated using triangulation. Finally, keyframes are selected, and the camera’s initial pose is estimated. If there are too many mismatches in the matches, it will lead to inaccurate initialization poses, adversely affecting subsequent localization and mapping. GMS-ATRANSAC is applied to the Initializer thread in the ORB-SLAM2 system, with an internal matching rate set to 40% for initialization experiments, and compared with the default initialization method of ORB-SLAM2.

**Figure 9:**
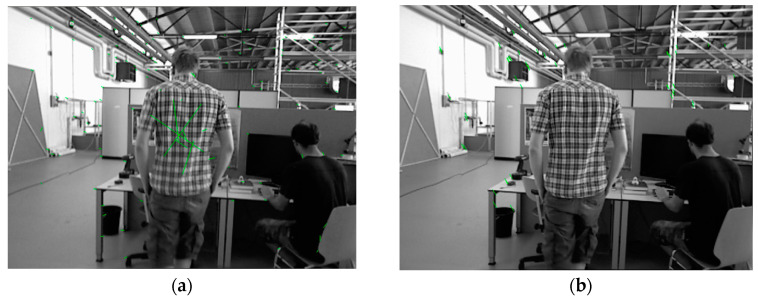
Comparison of the default initialization method (**a**) and the GMS-ATRANSAC initialization method (**b**) in ORB-SLAM2.

In Figure 9, both the left and right characters have some movement. The ORB feature points from the previous frame are matched with the current frame’s ORB feature points, and the motion relationships of the matched feature points are displayed by connecting them with green lines. Figure 9a shows the default initialization method of ORB-SLAM2. Due to the presence of many similar features on the checkered shirt, a large number of mismatches occur in the process of image feature matching, and the feature matches in the right character are not removed. The presence of a large number of mismatches and dynamic features leads to a low success rate of system initialization and inaccurate initial pose estimation. Figure 9b shows the initialization method using GMS-ATRANSAC, where the image feature matches on the characters have been completely removed.

#### 4.3.2. Tracking

After successful initialization, the Tracking thread of ORB-SLAM3 continuously tracks the camera pose in real time by detecting and matching features. An important step in the Tracking thread is the creation of keyframes, which are used to run the local map thread and loop closure detection thread. If there are too many dynamic feature matches in the keyframes, it can lead to relocation failure and inaccurate loop closure detection. Reducing dynamic features in keyframes is extremely important. To test the ability of GMS-ATRANSAC to remove dynamic feature mismatches in SLAM applications, GMS-ATRANSAC is integrated into the Tracking thread of ORB-SLAM3, with an internal matching rate set to 40%. If there are not enough feature matches, the minimum internal matching rate is increased. During the mismatch removal process, the keyframes are grouped into pairs, and GMS-ATRANSAC is used for feature mismatch removal within each pair. The removed match points are no longer used. The criteria for selecting keyframes remain unchanged.

**Figure 10:**
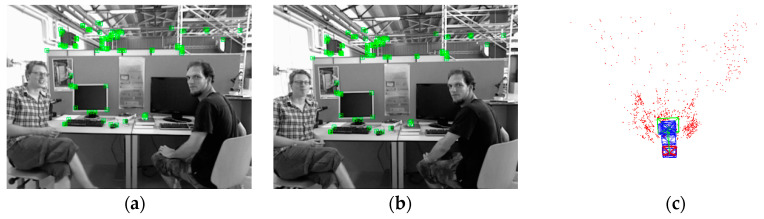
Adjacent keyframe feature points in the tracking thread of ORB-SLAM3 with the application of GMS-ATRANSAC in an indoor dynamic scene in (**a**,**b**). The depth point cloud map is shown in (**c**).

In Figure 10a,b, the movement of the person on the left includes upper body rotation, leg movement, and so on, while the head and body of the person on the right are moving. Figure 10c is a depth point cloud map of valid ORB feature points. The ORB-SLAM3 system, combined with GMS-ATRANSAC, effectively removes feature points in dynamic areas. Due to the intervals between keyframes in ORB-SLAM3, the movement of features in dynamic objects is more pronounced, and this algorithm can stably filter dynamic features. Filtering keyframes with features of dynamic objects enhances the accuracy of local map construction and loop detection.

## 5. Conclusions

This paper proposes an adaptive error threshold RANSAC (ATRANSAC) and combines it with the GMS. The GMS can quickly perform coarse screening of feature matches in a short time. Subsequently, ATRANSAC fine screening is performed after downsampling. The combination of GMS and ATRANSAC efficiently removes inter-frame feature mismatches, and the adaptive error threshold adjustment strategy empowers GMS-ATRANSAC with robust adaptability across various scenarios. Comparative experiments on image feature mismatch removal were conducted with the proposed method GMS-ATRANSAC, the classical method RANSAC, and the latest methods, GMS and GMS-RANSAC, in dynamic and static scenes of TUM and KITTI datasets. The experimental results indicate that GMS-ATRANSAC outperforms GMS in removing more mismatches. GMS-ATRANSAC reduces mean errors by an average of 29.4% and 32.9% compared to RANSAC and GMS-RANSAC, respectively; the variance of errors is reduced by an average of 63.9% and 58.0%, and the processing time is shortened by an average of 78.3% and 38%, respectively. Applying GMS-ATRANSAC in the initialization and tracking threads of ORB-SLAM2 and ORB-SLAM3 effectively removes mismatches and obtains more effective keyframes, demonstrating the effectiveness of GMS-ATRANSAC in visual SLAM. The GMS-ATRANSAC method brings new ideas and solutions to inter-frame feature mismatching removal in visual SLAM, contributing to the research and practical application of SLAM in dynamic scenarios.

In the following work, we will continue to improve GMS-ATRANSAC and homogenize the obtained matching results so that it can obtain more uniform and reliable image feature matching. Then, we will segment the image, such as the ground, pedestrians, vehicles, trees, etc., and store them after segmentation, and match the image features in the reliable segmentation area in each frame to obtain more accurate and uniform image feature matching. In addition, we will make relevant improvements to more existing SLAM algorithms, including visual SLAM and multi-sensor fusion SLAM, to increase the environmental adaptability of existing SLAM algorithms.

## Figures and Tables

**Table 1:** Mean error, error variance, and processing time of RANSAC, GMS-RANSAC (G-R), and the proposed algorithm (G-ATR) when processing 1500 ORB feature points.

Sequences	Metrics	RANSAC	G-R	G-ATR
TUM (Indoor static)	$e_{mean}$	1.489	1.655	1.236
	$e_{Var}$	0.843	0.642	0.353
	Processing time (ms)	94	25	12
TUM (Indoor dynamic)	$e_{mean}$	1.275	1.212	0.802
	$e_{Var}$	0.752	0.774601	0.278
	Processing time (ms)	95	34	22
KITTI (Outdoor dynamic)	$e_{mean}$	1.543	1.678	1.014
	$e_{Var}$	0.778	0.654	0.229
	Processing time (ms)	100	42	29

**Table 2:** Comparison of mismatching removal performance between the proposed algorithm and RANSAC and GMS-RANSAC when processing 1500 ORB feature points.

Sequences	$Imprv\left(e_{mean}\right)$ for RANSAC (%)	$Imprv\left(e_{mean}\right)$ for G-R (%)	$Imprv\left(e_{Var}\right)$ for RANSAC (%)	$Imprv\left(e_{Var}\right)$ for G-R (%)	$Imprv\left(Time\right)$ for RANSAC (%)	$Imprv\left(Time\right)$ for G-R (%)
TUM (Indoor static)	16.9%	25.3%	58.1%	45.0%	87.2%	52.0%
TUM (Indoor dynamic)	37.1%	33.8%	63.0%	64.1%	76.8%	31.0%
KITTI (Outdoor dynamic)	34.2%	39.6%	70.5%	64.9%	71.0%	31.0%
AVG.	29.4%	32.9%	63.9%	58.0%	78.3%	38.0%

## Data Availability

Data are contained within the article.
